# No good deed goes unpunished: the social costs of prosocial behaviour

**DOI:** 10.1017/ehs.2021.35

**Published:** 2021-07-21

**Authors:** Nichola J. Raihani, Eleanor A. Power

**Affiliations:** 1 Department of Experimental Psychology, University College London, 26 Bedford Way, London WC1H 0AP, UK; 2 Department of Methodology, London School of Economics and Political Science, Houghton Street, London WC2A 2AE, UK

**Keywords:** Prosocial behaviour, reputation, modesty, cooperation, partner choice, morality

## Abstract

Performing costly helpful behaviours can allow individuals to improve their reputation. Those who gain a good reputation are often preferred as interaction partners and are consequently better able to access support through cooperative relationships with others. However, investing in prosocial displays can sometimes yield social costs: excessively generous individuals risk losing their good reputation, and even being vilified, ostracised or antisocially punished. As a consequence, people frequently try to downplay their prosocial actions or hide them from others. In this review, we explore when and why investments in prosocial behaviour are likely to yield social costs. We propose two key features of interactions that make it more likely that generous individuals will incur social costs when: (a) observers infer that helpful behaviour is motivated by strategic or selfish motives; and (b) observers infer that helpful behaviour is detrimental to them. We describe how the cognition required to consider ulterior motives emerges over development and how these tendencies vary across cultures – and discuss how the potential for helpful actions to result in social costs might place boundaries on prosocial behaviour as well as limiting the contexts in which it might occur. We end by outlining the key avenues and priorities for future research.

## Graphical abstract

**Figure:**
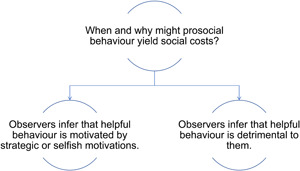

**Social media Summary:** Good deeds can result in social costs, particularly when observers suspect calculated or malign motives.

## Introduction

### Investing in costly cooperative behaviour can yield benefits

Humans are outliers in the extent and frequency of our cooperation, particularly when this occurs with non-relatives and in settings where the potential for direct reciprocal benefits is low (Raihani & Bshary, 2015; Rand & Nowak, 2013; Melis & Semmann, 2010; Bshary & Raihani, 2017; Henrich, 2017; Henrich et al., 2005; Boyd, 2017). Helping behaviour may be under positive selection because these costly investments allow an actor to signal something about themselves to observers (Barker et al., 2019). For instance, helpful acts can allow an individual to reveal their type (Gintis et al., 2001) or their intentions (McNamara & Barta, 2020; Roberts, 2020; Singh & Hoffman, 2021). Receivers can benefit from attending to such signals as this allows them to identify and preferentially assort with cooperative interaction partners (Barclay, 2016; Baumard et al., 2013; McNamara et al., 2008). Theoretical models have confirmed the evolutionary logic of reputation-based cooperation, via either indirect reciprocity (Kandori, 1992; Ohtsuki & Iwasa, 2006) or reputation-based partner choice (Fu et al., 2008; Li, 2014; Roberts, 1998, 2015). Experimental evidence from laboratory studies (including from non-humans; Bshary & Grutter, 2006) shows that the possibility of attracting partners is likely to be an important force for explaining the evolution and persistence of cooperation among non-relatives (Barclay & Barker, 2020; Barclay & Raihani, 2016; Raihani & Barclay, 2016; Sylwester & Roberts, 2010, 2013; Barclay & Willer, 2007; Tognetti et al., 2012; Van Vugt & Hardy, 2009; Fehrler & Przepiorka, 2016). Data from real-world settings (Hardy & Norgaard, 2016; Milinski et al., 2002a; Przepiorka et al., 2017; Raihani & Smith, 2015; Resnick et al., 2006; Yoeli et al., 2013) from many societies around the world (Bliege Bird et al., 2012; Bliege Bird & Power, 2015; Gurven et al., 2000; Lyle & Smith, 2014; Macfarlan et al., 2012, 2013; Macfarlan & Lyle, 2015; Power & Ready, 2018; Smith et al., 2003; Smith & Bird, 2000; von Rueden et al., 2019) also highlight the benefits that stem from being generous (or being seen to be generous): generous individuals are more likely to be helped themselves (Bliege Bird & Power, 2015; Gurven et al., 2000; Lyle & Smith, 2014; Macfarlan et al., 2012, 2013; Power & Ready, 2018), are healthier (Lyle & Smith, 2014) and have more and healthier offspring (von Rueden & Jaeggi, 2016).

### But benefits do not always follow from prosocial actions

It is perhaps less well appreciated that cooperative actions can result in social costs rather than benefits. By revealing their cooperative intentions, individuals might leave themselves vulnerable to exploitation by others (e.g. Bereczkei et al., 2015), particularly when players only expect to interact once (Roberts, 2020). Yet signalling prosociality can also invite hostility and competition from others. For instance, individuals often refuse offers of help (Ackerman & Kenrick, 2008), dislike those who offer help (Fisher et al., 1982) and may find overly generous individuals unattractive (Bhogal et al., 2020). Those who perform morally laudable behaviours in public also risk being accused of ‘virtue signalling’ (Levy, 2020). In laboratory social dilemma games, people's antipathy towards prosocial individuals often manifests as antisocial punishment (Herrmann et al., 2008; Sylwester et al., 2013). Although people generally tend to direct punishment at the free-riders in the group (Falk et al., 2005; Fehr & Gächter, 2000, 2002; Johnson et al., 2009; Nikiforakis & Engelmann, 2011), a persistent fraction of players in social dilemma games sacrifice some of their personal endowment to fine cooperative co-players (Herrmann et al., 2008; Raihani & Bshary, 2019; Sylwester et al., 2013). Variants of such games that allow players to vote to evict members of the group rather than to financially punish them find similar results: the most cooperative group members are evicted as often as the worst free-riders (Parks & Stone, 2010). The tendency to dislike or to disparage prosocial or morally laudable others has also been studied under the banner of do-gooder derogation (Bai et al., 2019; Bolderdijk et al., 2018; Minson & Monin, 2012; Monin, 2007; Sparkman & Attari, 2020a; Zane et al., 2016) – a phenomenon whereby individuals who perform morally laudable actions (e.g. refraining from eating animal products or defending minority groups) are derogated by peers. This tendency to dislike generous or moral others has also been found in children as young as 8 years old (Tasimi et al., 2015). Although it is clear that good deeds can result in social costs, less work has systematically explored when and why these costs arise. We attempt to do so in this paper.

## When and why might helpful signals yield social costs?

We propose that generosity is least likely to yield benefits and most likely to result in social costs in two, non-mutually exclusive scenarios:

1. observers infer that helpful behaviour is motivated by strategic or selfish motives;
2. observers infer that helpful behaviour is detrimental to them.

In what follows, we use the word observer to mean ‘someone who learns about the prosocial action of another individual’. In principle, the observer could learn because they are the recipient of the prosocial act or because they are an uninvolved bystander. We use the word observer to denote both possibilities. We expand on these predictions below.

### Observers infer strategic or selfish motives

Praiseworthiness depends on attributing intentions to actors (Anderson et al., 2020; Bénabou & Tirole, 2006; Pizarro et al., 2003; Siegel et al., 2017), but the motives underpinning helpful actions are not visible to an observer. Instead, motives are typically inferred based on the helper's behaviour (e.g. the type and extent of help offered), any emotions revealed during the behaviour and the context (including the sociocultural setting, see Barrett et al., 2016; Henrich, 2020) in which the behaviour occurs.

Motives and outcomes do not always cohere: bad outcomes can stem from benign intentions, and good outcomes can stem from malign or selfish intentions. In principle, helpful actions can stem from proximately ‘altruistic’ motivations – that is, a genuine desire to help others (e.g. Andreoni, 1990; Batson et al., 1981; Bénabou & Tirole, 2006; Harbaugh et al., 2007). Nevertheless, at least part of the motive underpinning helpful actions may also be strategic or self-interested. For example, in experimental Dictator and Ultimatum games, a standard result is that people typically offer more money to a partner in the Ultimatum than in the Dictator game (Camerer, 2011). This result can be understood in light of the strategic nature of the Ultimatum game (where an offer that is perceived to be too low can be rejected by the responder) compared with the non-strategic nature of the Dictator game (where receivers must accept whatever share the dictator sends). Similarly, people often behave more generously in situations where their behaviour can be observed (e.g. Andreoni & Petrie, 2004; Bereczkei et al., 2007; Harbaugh, 1998; Hoffman et al., 1994; Yoeli et al., 2013) and can affect how others may treat them in future (Barclay & Willer, 2007; Milinski et al., 2002b; Semmann et al., 2004; Sylwester & Roberts, 2010, 2013), which also indicates that prosocial actions are influenced by strategic concerns.

These patterns hint at the complexity of decoding the motives underpinning generosity: some helpful behaviour may well be motived by pure altruism, but it is clear that humans are also sensitive to the possibility of personal gains and adjust their investments in helpful behaviour accordingly (Semmann et al., 2004), even from a young age (Warneken et al., 2019). Observers (and recipients) of prosocial acts therefore face the difficult task of reconciling the actions they observe with a range of possible underlying motives. Some of these motives are likely to be more indicative of the helper's underlying cooperative disposition than others.

We propose that observers will be more likely to infer that help stems from strategic rather than altruistic motives when helpful actions appear to be aggrandising, for example if they are performed in public or are actively broadcast to others by the helpful individual. Several studies have shown that individuals who advertise their good deeds (Berman et al., 2015; De Freitas et al., 2019; Siem & Stürmer, 2018) or who know that their good deeds will be visible to others (Gambetta & Przepiorka, 2014) risk being perceived as less generous by observers (Srna et al., 2017). Similar results have been obtained in the context of corporate social responsibility (CSR). Specifically, consumers are more sceptical of CSR activities when they suspect that these deeds stem from profit-seeking rather than benevolent motives (Berge & Arshad, 2020; Chernev & Blair, 2015; Sen et al., 2016) and they downgrade their assessment of CSR initiatives when they learn of these from the company's advertising rather than from other sources (Yoon et al., 2006).

In general, positive acts are more likely to result in reputation benefits when observers can infer that the helper is motivated by empathic concern or that they are eager and willing to help. Indeed, the reputation consequences of actions may be affected more by the emotions attributed to the actor than to the outcome of the action itself (Yudkin et al., 2019). People rely more on emotions than on outcomes when judging helpful individuals because emotions provide a more reliable cue as to the person's underlying character or disposition (Barasch et al., 2014; Frank, 1991; Hoffman et al., 2015; Levine et al., 2018; Reed et al., 2012) or to the value they place on the relationship (Aktipis et al., 2018; Ames et al., 2004; Delton & Robertson, 2016; Frank, 1991; Hoffman et al., 2015) – and are therefore a more reliable guide to how the person might behave in future (see also Hirshleifer, 1987; Singh & Hoffman, 2021). For instance, people who perform prosocial actions but display reluctance or negative emotions while doing so are unlikely to accrue reputation benefits as the negative emotional signal nullifies the positive act (Ames & Johar, 2009; Carlson & Zaki, 2018; Krull et al., 2008). Similarly, people who express mixed motives for helping or who help without expressing empathy are also less positively evaluated by others (Erlandsson et al., 2020). When evaluating the moral consequences of charitable donations, people rate those who donate time as being more praiseworthy than those who donate money (even though people also acknowledge that time donations are often less efficient; Johnson & Park, 2019). This evaluative premium on time arises in part because people perceive time donations as signalling a greater emotional investment in the cause and therefore a better indicator of the donor's underlying character.

The idea that observers try to discern a helper's underlying character or disposition when evaluating prosocial acts is also evident in the negative reaction to hypocrisy or inconsistent behaviour (Effron et al., 2018; Jordan et al., 2017). For example, in one vignette-based study, individuals who signalled moral virtue (by criticising others) but subsequently behaved immorally themselves were more negatively judged than individuals who admitted their moral failings (Jordan et al., 2017). Similar reactions are observed in the domain of CSR and can help to explain why these activities sometimes backfire: if companies with bad reputations engage in CSR, consumers are more likely to infer that these socially responsible actions stem from a desire to whitewash a bad reputation (Yoon et al., 2006). CSR activities that seem at odds with the broader mission of the company can also negatively impact brand evaluations (Silver et al., 2021; Torelli et al., 2012). Indeed, companies that emphasise the business case for CSR tend to be more positively evaluated than those who emphasise the moral case because emphasising the business case allows the company to avoid accusations of moral hypocrisy (Hafenbrädl & Waeger, 2019). A key point here seems to be that being upfront about the underlying reasons (or potential benefits) associated with performing prosocial deeds can quell the suspicion these deeds might otherwise arouse in some cases.

Relatedly, observers might also suspect that helping stems from strategic rather than altruistic motives if the helper deliberates or assesses the costs before deciding whether to help (Hoffman et al., 2015). Participants who cooperate without knowing the cost of doing so (Jordan et al., 2016) – or who make their decision quickly (Critcher et al., 2013; Evans & van de Calseyde, 2017) – are perceived as more prosocial or trustworthy by others – as are those who make deontological, rather than utilitarian, decisions when faced with moral dilemmas (Everett et al., 2016). Similarly, individuals who donate based on the cost-effectiveness of the charity, rather than based on the degree of empathy they feel for the beneficiaries, are evaluated less positively by others (Montealegre et al., 2020). Subjects who cooperate in seemingly one-shot encounters are also perceived as more trustworthy than those who cooperate in encounters that they know will be repeated (Johnsen & Kvaløy, 2016), perhaps because cooperating in interactions that are known to be repeated is more indicative of strategic motives.

The appearance of strategic, calculating decision-making is particularly costly in long-term reciprocal exchanges (‘friendships’), when an effort to quickly reciprocate and erase a debt may reduce what could be seen as an open-ended cooperative partnership to a disinterested series of transactions (Bourdieu, 1990; Silk, 2003). The logic of tit-for-tat in a long-term relationship can be damaging because it implies low interdependence (Aktipis et al., 2018; Kim et al., 2019; Roberts, 2005). This can explain why subjects tend to repay favours from strangers more quickly than those from friends (Boster et al., 1995) and can take offence – and judge friendships as less close – when friends immediately reciprocate kind acts (Shackelford & Buss, 1996; Silk, 2003) or give gifts with the expectation of adulation or return favours (Lee, 1969).

Motives might also be inferred based on whether the helper materially benefits from their actions. For example, donors to charity are perceived as less charitable if they donate to a cause to which they have a personal connection (Lin-Healy & Small, 2012), and advocates for a charitable cause are perceived as less sincere and garner fewer donations for the cause if the advocates reap financial benefits from this association (Barasch et al., 2016). In corporate settings, organisations that perform good deeds are given less credit if doing good helps their bottom line (Makov & Newman, 2016; Newman & Cain, 2014), an effect known as ‘tainted altruism’ (Newman & Cain, 2014). In keeping with this view, people more positively judge helpful actions when these involve a degree of personal sacrifice (Bigman & Tamir, 2016; Carlson & Zaki, 2018; Johnson, 2018; Kawamura et al., 2020; Nelissen, 2008; Visserman et al., 2018). Indeed, in the context of charitable donations, people are more sensitive to personal sacrifice than social benefit when evaluating prosocial behaviour (Johnson, 2018). This increased perception of costs, and so an assumed lesser material benefit, can explain why lower status helpers are sometimes perceived in a more positive light. A vignette study found that lower class donors (measured in terms of occupation and salary) were more likely to be seen as prosocial, compared with higher class donors, when giving the same (either relative or absolute) donation to a charity, as the lower-status individuals were seen as more authentically motivated (Yuan et al., 2018).

### Observers infer that helping behaviour is detrimental to them

Although prosocial acts are typically interpreted as yielding benefits to others, this may not always be the case. Instead, observers might sometimes perceive (accurately or not) that a prosocial act is likely to harm, rather than to benefit, them. For this reason, the reputation consequences of helping are also likely to depend on who is watching: helpful acts might improve your reputation in the eyes of some individuals but not others. Broadly, it seems plausible that observers who incur costs or infer that they are disadvantaged as a consequence of the helpful actions of another individual will be more likely to evaluate the helpful individual negatively. We outline some specific cases below.

One of the most obvious ways that observers might incur costs from the actions of helpful individuals is due to social comparison. A good reputation is, by definition, a positional good – a person's reputation is ‘good’ in relation to the reputations of other individuals to whom that individual is compared (Barclay, 2011, 2013, 2016; Samu et al., 2020). Prosocial actions that improve one person's reputation (or can be construed as potentially doing so) can therefore provoke competitive responses from those whose reputation may suffer by comparison (e.g. Herrmann et al., 2019; Macfarlan et al., 2012; McAndrew & Perilloux, 2012; Pleasant & Barclay, 2018; Raihani & Smith, 2015; Sylwester & Roberts, 2013). Similarly, if status hierarchies are formed in part on the basis of patronage and largesse, then one person's generous acts may have the effect of lowering others’ relative positions within that hierarchy. Gift-giving or hosting large feasts, for example, may be arenas for status competition and the demonstration of power and authority (Boone, 1998); such acts may therefore invite negative reactions from those displeased with how they will look in comparison. Studies exploring intergroup relations have shown that help directed from higher to lower status groups can be resisted as it can be interpreted as an attempt to assert or maintain dominance. For example, when asked to imagine receiving help from an out-group, Israeli Arabs were more likely to judge help from Israeli Jews as being directed at reinforcing existing power dynamics (Halabi et al., 2016; Nadler & Halabi, 2006). Cross-cultural work using economic games also suggests that people resist generous overtures that can be interpreted as attempts to establish dominance (Henrich et al., 2005).

Antisocial punishment and other hostile reactions to cooperators observed in social dilemmas are thought to be prompted at least in part by social comparison. For example, in a public goods game, where players could vote to evict one player from the group after each round, players often voted to evict the most generous member from the group (Parks & Stone, 2010). When asked to justify these decisions, participants frequently referenced their own reputation, for example ‘No one else is doing what he does. He makes us all look bad’ and ‘Next to Person Blue I look like a bad guy, but I don't think I am’. Other studies have also shown that excessive generosity is judged unfavourably and may even be punished (Bahry & Wilson, 2006; Irwin & Horne, 2013; Kawamura & Kusumi, 2020). In contrast, antisocial punishment of overly cooperative individuals is rare when the punisher is a third party, rather than a player who was involved in the public goods game (Bone et al., 2014). Since the third party cannot contribute to the public good, their reputation as a generous or cooperative individual is not affected by the investments of the other players. The fact that uninvolved parties do not tend to antisocially punish therefore supports the idea that hostile reactions to helpful individuals are mediated by social comparison. In a more recent study, Pleasant and Barclay (2018) showed that antisocial punishment in a public goods game was most prevalent when individuals were being evaluated as a partner for a subsequent Trust Game, which further supports the idea that antisocial punishment operates via concern for reputation and relative standing. As with antisocial punishment, experimental studies have shown that do-gooder derogation frequently stems from competitive motives – because people who appear to be morally superior can prompt less moral individuals to evaluate themselves more negatively (Minson & Monin, 2012; Monin, 2007; Monin et al., 2008).

Observers might also negatively evaluate helpful individuals when the helper's and the observer's goals are misaligned (Melnikoff & Bailey, 2018). For example, people prefer moral traits in others (such as mercifulness, loyalty and trustworthiness) only when these traits are conducive to their current goal. In scenarios where it would benefit these individuals to interact with merciless, fickle or dishonest others, then these traits are more positively evaluated (Melnikoff & Bailey, 2018). A similar discrepancy has been observed in moral dilemmas, such as the trolley problem, where people prefer deontological agents when choosing social or romantic partners but prefer consequentialist (utilitarian) individuals when electing political leaders (Everett et al., 2018).

Another way in which a helper's goals might be misaligned with an observer's is when the help offered to one person is traded off against the help that can be offered to another (Law et al., 2019). For example, in Western cultures, helping strangers is typically perceived as being a morally positive thing to do (Henrich, 2020; McManus et al., 2020). However, even in a sample of US residents, people who help strangers instead of helping family were generally judged more negatively (McManus et al., 2020), indicating that helpers’ reputations are formed on the basis of who is helped and the trade-offs involved. Related to the above, helpful acts can sometimes threaten the observer's existing relationship with the helper – for example by calling into question the closeness of the relationship (Law et al., 2019) and thereby potentially sparking jealousy (Krems et al., 2021).

Helpers might also be judged more negatively when the help disadvantages the observer's in-group or is directed towards the observer's out-group (cf. Fessler et al., 2015). For example, one study measured participants’ reactions to another individual who confronted discrimination, involving both sexism and racism as a function of whether the confronter was part of the disadvantaged group (e.g. the confronter is also a woman in the sexism example) or part of the advantaged group (e.g. the confronter is a man in the sexism example; Kutlaca et al., 2020). In this study, participants were more likely to judge that the confronter had over-reacted if both the participant and the confronter were members of the same advantaged in-group. Similarly, whistleblowers within organisations are seldom rewarded for their actions and are often more likely to be bullied than those who keep quiet (Bjørkelo et al., 2011). The evaluation of any particular ‘helpful act’ must, then, lie in the eye of the beholder.

## Reputation management strategies in humans

Given the potential social costs associated with performing overtly generous or moral actions, it is not surprising that people manage their reputations in quite sophisticated ways, for example by refraining from doing good deeds in public or by actively hiding their good deeds from others. While many studies have shown that people are more cooperative when their behaviour is public (e.g. Andreoni & Petrie, 2004; Bekkers & Wiepking, 2011; Bereczkei et al., 2007; Bohnet & Frey, 1999; Piazza & Bering, 2008; Yoeli et al., 2013), other studies report mixed or contrasting results. Notably, a recent meta-analysis found that although there is a small and statistically significant positive effect of observability on prosocial behaviour, the effect is highly heterogeneous and context-dependent (Bradley et al., 2018). For example, public recognition can decrease people's willingness to donate blood (Shi, 2011) or to donate to charity (Denis et al., 2020; Savary & Goldsmith, 2020; Simpson et al., 2018). Experimental studies have shown that individuals feel uncomfortable when they learn that they are morally superior to their peers (Cowgill, 2017) or when their moral actions are highlighted to others (Wang & Tong, 2015) and these negative feelings might prompt individuals to hide their good deeds from others. For instance, when making charitable donations online, people often hide their donations from others by making them anonymously (*Big Charitable Gifts*, 2020; Imada, 2020) and the tendency to donate anonymously (either by hiding their name or the amount donated) is higher when the donation is especially generous (Mokos & Scheuring, 2019; Peacey & Sanders, 2014; Raihani, 2014). Similar results have been obtained in experimental settings (Jones & Linardi, 2014), although we note that most of these studies involve WEIRD (Henrich et al., 2010) populations and it remains an open question as to whether these patterns would generalise to different cultures.

People also seem to be sensitive to the possibility that their good deeds will reveal self-interested intentions, and often work to prevent such inferences from being made. For example, when donating to charity, people are more likely to perform charitable actions that are perceived as being painful or difficult (Olivola & Shafir, 2013), perhaps to lessen any suspicions of self-interest in ostensibly altruistic behaviour. Similarly, time donations are viewed as more morally praiseworthy than money donations and – as we might expect – people often prefer to donate time than money, even when the latter could have a larger impact (Johnson & Park, 2019). In studies where people could cooperate in a calculated or uncalculated manner (by revealing the cost associated with cooperating), people are more likely to opt for uncalculated cooperation when they know their decision is observed by others (Jordan et al., 2016). Similarly, hunters, especially of large game, may practice ‘pecuniary distancing’ by actively avoiding having control over the divvying up and sharing of their catch, so that others cannot infer selfish intent (Bliege Bird & Power, 2015).

Individuals also manage their prosocial reputation in other ways, for example by selectively investing in subtle, rather than ostentatious signals (Bliege Bird et al., 2018). The very subtlety and discreetness of acts may help ensure that they are seen as generous and well intentioned: when no audience is there to provide a reputational boon, it should be much harder to infer that the intention was one of self-aggrandisement. Helpful acts which are experienced and observed only (or primarily) by the intended recipient may then do much to assure that person of the giver's commitment to their continued relationship.

Recent theoretical work has also shown how obscuring or ‘burying’ costly signals can also be under positive selection (Hoffman et al., 2018). This occurs because buried signals come with a tag that identifies them as having been buried (e.g. we might know whether our wealthy friend chose to donate anonymously on a fundraising platform). This allows the fact that the signal has been buried to act as a signal in itself. Although initial work found that only the ‘best’ signallers would evolve to bury their signals, a subsequent analysis found that a more universal modesty norm can also spread through populations (Quillien, 2019). Empirical studies support these models, by showing that modesty begets modesty and that modest actions are likely to be socially learned. For example, in one study of online charitable giving, the presence of anonymous donations encouraged subsequent donors to anonymise their own donations (Burtch et al., 2016).

In general, we suggest that concern for reputation might often manifest as behaviour aimed at preventing reputational damage, rather than behaviour aimed at actively improving reputation. This is partly because, as the evidence above indicates, prosocial acts do not always result in social benefits. Not only that, but there is a further asymmetry in the social consequences of blameworthy and praiseworthy acts: there is often more to be lost from being seen as blameworthy than to be gained from being seen as praiseworthy (reviewed in Anderson et al., 2020). For example, breaking a promise is viewed as morally contemptible, but over-delivering on a promise carries no reputation benefits relative to simply keeping it (Gneezy & Epley, 2014). Similarly, moral judgements of selfish actions are moderated by the magnitude of the selfishness (more selfish actions are judged morally worse), but the converse is not true for selfless actions: the benefits of behaving prosocially do not scale linearly with the magnitude of the prosocial deeds (Klein et al., 2015; Klein & Epley, 2014). Other work has shown that blameworthiness judgements are often more extreme than praise judgements (Guglielmo & Malle, 2019) and more sensitive to outcomes. Given all this, the risk of a generous act resulting in social costs may weigh more heavily in people's minds than the potential benefits that could stem from it.

## The cognitive underpinnings of managing and evaluating reputations

There is clearly more to reputation management – and impression formation – than the simple performance of prosocial actions to reap social benefits. Instead, when forming an impression of others, we have to be able to infer the motives and intentions that accompany positive actions. When managing our own reputation, we also have to be able to infer how our own actions – and potentially divergent perceptions of our intentions – will affect how others form and update their assessments of us. The ability to manage one's own reputation and to evaluate the reputations of others relies on ‘epistemic vigilance’ (Sperber et al., 2010) – a suite of cognitive abilities which probably evolved to help us discern reliable from unreliable communication. These abilities emerge at different stages of development (Banerjee, 2000; Banerjee et al., 2020; Engelmann & Rapp, 2018; Silver & Shaw, 2018; Sperber et al., 2010) and show differential expression in reputation-based cooperation across societies (Amir & McAuliffe, 2020; Cowell et al., 2017).

A key feature of epistemic vigilance is the ability to infer intentions that appear to be at odds with observed actions (e.g. *The actions suggest X but the motive is Y*). This form of intention attribution is distinct from and computationally more demanding than the simpler version of intention attribution (likely to be present in non-human apes and very young children; Tomasello et al., 2005), where observers impute intentions that cohere with a target's observed behaviour. The development of scepticism – where we suspect that an agent is trying to present themselves in a favourable light – involves doing something even more sophisticated, however: we may intuit not only that A's intentions are at odds with their actions, but also that A would like us (or other observers) to form a false belief about their intentions (e.g. *A wants me to believe their motive is X when I suspect in fact it is Y*). This latter awareness involves reasoning at a higher meta-representational level (Sperber et al., 2010), which may account for its relatively late emergence during childhood (Heyman et al., 2007; Heyman et al., 2014).

Children start to manage their own reputation – and to evaluate the reputations of others – in early childhood (Banerjee et al., 2020; Engelmann & Rapp, 2018; Silver & Shaw, 2018). The ability to form impressions of others seems to emerge earlier in development than the ability to manage one's own reputation: indeed, some studies have even found that pre-verbal infants evaluate others on the basis of helpfulness (Hamlin, 2013; Hamlin et al., 2011; but see Salvadori et al., 2015; Schlingloff et al., 2020 for failed replications). Whether or not the ability to evaluate others emerges in preverbal infants, this ability does seem to be reliably evident by early childhood. By the time they are around two and a half years old, children can discriminate a nice partner from a mean one, only on the basis of observation, and prefer to interact with the nice partner (Herrmann et al., 2013). Children as young as three years old expect that individuals will share information that presents them in a good light, for example, by revealing their positive performance on a puzzle but withholding their failures (Hicks et al., 2015); and by the age of five, children begin to gossip, offering their opinion on the character of others to help a third party to decide who to interact with (Engelmann et al., 2016). Four-year-olds appreciate that others will lie to benefit themselves and, by the time they are six, children distinguish between self-interested lies and lies intended to benefit others (Banerjee et al., 2020; Mills & Keil, 2005). In one study, children were asked to evaluate a protagonist who either plagiarised someone else's story (and took the credit for themselves) or who lied to give the credit for their own story to another child (Shaw & Olson, 2015); children were more likely to evaluate the person who stole the credit negatively – and to evaluate the person who gave credit away positively.

In contrast to the early emergence of impression formation, children do not begin managing their own reputation until they are around five years old. By this age, children (but not chimpanzees) are sensitive to the presence of an audience when making prosocial decisions (Engelmann et al., 2012) – and give more when their decisions will be revealed to others (Leimgruber et al., 2012). Nevertheless, before middle childhood (around the age of 8) children tend not to reason explicitly about how their actions will affect other individuals’ impressions; rather, reputation management in young children has been referred to as ‘implicit’ and may overlap with what has been called ‘audience effects’ in non-human species. By middle childhood, children start to explicitly reason about how their actions will affect other people's impressions of them (Banerjee et al., 2020; Engelmann & Rapp, 2018)– and it is at this point that children also start to understand that other individuals may do the same (Heyman et al., 2007, 2016; Heyman et al., 2014). For example, although young children do not differentiate between acts of generosity that are performed in public vs. those that are done in private, by the time they are eight years old, children reliably rate private acts of beneficence as more morally praiseworthy (Heyman et al., 2016; Heyman et al., 2014). In middle childhood, children also begin to exhibit scepticism about self-serving claims of prosociality (Amemiya et al., 2020; Heyman et al., 2007; Heyman et al., 2014), although this ability continues to develop over this period. For example, older children (aged nine to 10) appreciate that self-promotional statements are more morally permissible when they can also be construed as implicit offers of help (Amemiya et al., 2020).

At the same time as children start to appreciate that others strategically manage their reputation – and develop a sense of scepticism about people who use self-promotional strategies – they also begin to understand the benefits of modest actions and to evaluate modest others as more morally praiseworthy (Banerjee, 2000). The importance of modesty – and the extent to which children both strive to appear modest and approve of modesty in others – varies cross-culturally, being more strongly emphasised in East-Asian than in Western societies. For example, in a study involving Japanese and American schoolchildren (aged seven to 11 years), the tendency to approve of modest individuals (who lied about their prosocial actions) increased with age in both groups (Heyman et al., 2011). Nevertheless, the Japanese children were also more likely to disapprove of those who truthfully accepted the praise for their good deed, a pattern that was not observed among the American children. A more recent study comparing self-effacement in a sample of Chinese and Canadian children of the same age found that the tendency to eschew moral praise increases with age – but that this effect was more pronounced among the Chinese children (Fu et al., 2016). These and related studies strongly suggest a role for social learning in the development of modesty traits (see also Banerjee, 2000) and emphasise the fact that modesty norms (and associated reputation management strategies) are likely to vary cross-culturally (Genyue et al., 2011; Kanagawa et al., 2001; Yamagishi et al., 2012). Cross-cultural evidence generally suggests that responsiveness to local norms solidifies in middle childhood, and that this can precipitate important variation in prosocial behaviour (Amir & McAuliffe, 2020; Blake et al., 2015; Cowell et al., 2017; House et al., 2013, 2020a, b).

In sum, several cognitive abilities are implicated in even basic reputational models of cooperation – including the ability to mind-read and attribute intentions and motives to others, to be sensitive to the local norms in order to judge whether behaviours are praiseworthy or contemptible, and to infer how others might perceive us based on our actions and their own perceptions of our hidden intentions. These skills do not all emerge at a single set point in development, but are instead honed over childhood and into adolescence, are responsive to local cultural norms, and are generally highly contingent and situational.

## Larger-scale consequences of perverse reputational incentives

Considering the social costs incurred by prosocial individuals and organisations has broader implications for the emergence and persistence of cooperation within groups, as well as for the effectiveness of these altruistic efforts. Many of the social interactions we face can be broadly described as social dilemmas: mutual cooperation yields the best collective payoffs, but individuals can derive a larger short-term benefit from acting selfishly. Finding ways to resolve such social dilemmas – and to encourage mutual cooperation – is both theoretically and practically important (e.g. Raihani & Aitken, 2011; Ostrom et al., 1999; Van Vugt, 2009; Penn, 2003). A key insight from experimental economics and social psychology is that many people are conditional cooperators (Fischbacher et al., 2001). Under this view, good deeds can spark further cooperation (Gino et al., 2009; Jordan et al., 2013; McAuliffe et al., 2015; Pfeiffer et al., 2005; Raihani & Hart, 2010) and uncooperative acts prompt reciprocal defection, potentially leading to the unravelling of cooperation (e.g. Raihani & Hart, 2010). Although we do not want to challenge the notion of conditional cooperation, we also want to emphasise that cooperative individuals can also sometimes undermine the emergence of large-scale cooperation. For example, people often hold negative stereotypes of socially conscious individuals – and these perceptions can dissuade others from cooperating in these domains (Bashir et al., 2013; Fruhen & Flin, 2015). The investments of prosocial individuals can also deter individuals who might otherwise cooperate, either because they do not feel the same degree of commitment to the cause as these moral minorities (Kurz et al., 2020) or because they fear moral reproach if their own efforts fall short by comparison (Howe & Monin, 2017; Sparkman & Attari, 2020b; Stouten et al., 2013).

Moreover, negatively evaluating prosocial individuals and companies can act as a further obstacle to achieving large-scale cooperation because these social costs act as an additional disincentive to pay the costs associated with cooperative behaviour. For example, people and companies whose actions yield personal *and* social benefits are typically evaluated more negatively than counterparts whose actions yield no social benefits at all (Makov & Newman, 2016; Newman & Cain, 2014). By disparaging people who personally benefit from performing good deeds – or by enacting preferences against for-profit, for-good companies – people might disincentivise those who might otherwise invest in socially beneficial activities.

The special credit we afford to deeds seemingly motivated by ‘pure’ emotions compared with those that stem from ‘deliberative’ calculation can also have unwanted large-scale consequences. For instance, despite the large sums that are collectively donated to charity (around $400 billion per year in the US), many people do not prioritise ‘effectiveness’ in the decision of which charity to support (Caviola et al., 2020). Instead, people support charities based on their subjective preferences (Berman et al., 2018), being more likely to donate to disaster-relief campaigns than to charities that aim to resolve recurring problems (Caviola et al., 2020) or donating to causes that are associated with single identifiable victims rather than those which will help larger numbers (Jenni & Loewenstein, 1997). Ineffective altruism can nevertheless make sense in light of its reputation consequences. For example, those who deliberate over which charity to support are evaluated less positively than those who use empathy as their moral compass (Montealegre et al., 2020) and, despite the fact that donating to charities in lower-income countries is more effective than donating to more local causes (Caviola et al., 2020), people often disapprove of those who forego opportunities to help at a local scale in favour of helping more distant others (Law et al., 2019; McManus et al., 2020). More generally, the social rewards that flow from prosocial acts tend to be sensitive to the costs and observability of the good deeds and typically do not scale with the efficacy of the helpful act, which can explain why people engage in ineffective helping (Burum et al., 2020).

The tendency to seek ulterior motives behind ostensibly prosocial actions can also have effects on self-assessments of our own helpful deeds, which subsequently affect the tendency that we will repeat them. Specifically, it appears to be easier to maintain a positive self-image and to derive a warm glow from giving when we can be assured that our own motives were not corrupted by extrinsic incentives (Gneezy et al., 2012; Savary et al., 2020; Savary & Goldsmith, 2020; Small & Cryder, 2016). It is now well established that external incentives for prosocial behaviour – such as thank you gifts or financial incentives, like tax breaks – frequently reduce people's willingness to perform these prosocial acts (Chao, 2017; Gneezy & Rustichini, 2000; Mellström & Johannesson, 2008; Moussaoui et al., 2019; Newman & Jeremy Shen, 2012; Savary et al., 2020; Shi, 2011). These negative effects can be ameliorated by giving people the opportunity to forego the external incentive (Mellström & Johannesson, 2008) – and other work indicates that people often engage in such ‘motivational laundering’: foregoing an external incentive to remove ambiguity about the motives underlying their charitable actions (Kirgios et al., 2020).

## Conclusions and future directions

In this final section, we outline some specific directions for future research, focusing on research gaps and open questions. One key hypothesis stemming from this review is that people might often seek to avoid social costs rather than to garner social benefits – particularly when it comes to reputation management in daily life. Predictions stemming from this hypothesis cannot easily be tested with experimental paradigms that constrain the ways in which individuals are allowed to manage their reputation (for example, using experiments that only allow individuals to signal prosociality to attract interaction partners). Instead, we believe that a more naturalistic, observational approach as used in studies of animal behaviour would be helpful to address this hypothesis. The rich insights from ethnography and participant observation will surely have a role here. Alongside this, new technologies allow for experience sampling methods and have been used to great effect to address other aspects of social behaviour in daily life (e.g. Molho et al., 2020; Weiss et al., 2020). Vignette studies, wherein individuals are presented with a situation and give their reaction or outline potential courses of action may also provide a promising avenue (cf. Mathew & Boyd, 2014). Such methods could be used to measure the various ways that people manage their reputations and form and update impressions of others and to test the idea that avoiding social costs takes precedence over seeking reputation benefits.

Another key theme coming out of the existing literature on reputation-based cooperation is that both reputation management and impression formation rely on a suite of cognitive abilities, which seem to emerge during middle childhood. This is now a burgeoning area of research and great strides are being made in identifying the cognitive abilities that scaffold reputational strategies. However, it is still unclear which cognitive mechanisms are key to these abilities and how exactly these abilities are attained. Specifically, it is not clear whether the cognition supporting sophisticated forms of reputation-based cooperation emerges at relatively fixed points in development or whether the abilities have to be culturally learned and practised during childhood (Heyes, 2019; Luhrmann, 2011). We also know rather little about how individual variation in socio-cognitive abilities affects reputational strategies. For example, do individuals vary in their ability to both manage their own reputation and to form accurate impressions of others; and, if so, where does this variation come from? It may also be fruitful to explore metacognitive accuracy – specifically whether individuals are able to judge how signalling their prosocial deeds might be interpreted by others, as well as the factors that predict performance in this domain. We also note that previous studies have documented societal variation in the tendency to account for mental states when judging harmful outcomes (Barrett et al., 2016; Curtin et al., 2020): in countries that have stronger kin-based institutions, people are less likely to take an actor's mental state into consideration when forming moral judgements. As we have argued, intention attribution is also pivotal in determining how people response to actions with beneficial outcomes but much of this evidence is derived from Western societies. A clear research priority is therefore to determine whether consideration of mental states when judging beneficial acts varies in a similar manner around the world.

It is also apparent that the content of the norms we use to guide our behaviour and form impressions of others varies hugely around the world. There is a clear need to understand how cognition and culture exert their independent and interacting effects on reputational strategies in humans; and this field would benefit from theoretically informed work addressing how and why such variation arises. For example, although it seems apparent from the studies above that modesty norms are more strongly endorsed in East Asian (compared with Western) societies, it is not especially clear why this is the case: the observation that modesty is more highly prized in some societies as compared with others falls short as an explanation as it simply restates the puzzle.

Crucial to any explanation of cross-cultural variation in the importance of reputation and social costs/benefits will be an understanding of the nature and structure of social relations: for example, the extent of people's interdependence (Aktipis et al., 2018; Roberts, 2005), their embeddedness in dense kin networks (Enke, 2019; Henrich, 2020; Schulz et al., 2019), their ability to extricate themselves from existing relationships (Jackson et al., 2012) and the importance of social relations to future generations (Bowles et al., 2010; Borgerhoff Mulder et al., 2009). Such variation in the form and salience of social relations, which can stem from differences in ecology and mode of production (Bowles et al., 2010; Lamba & Mace, 2011), as well as historical contingency and cultural norms, should help account for variations in social norms and institutions around the world. Comparative, cross-cultural work that captures these aspects of social structure (whether through social network collection or field experiments that retain elements of people's social network position and connections) is needed to help us understand these points.

Finally, just as there is a need to explain variation across societies, so too is there a need to explain variation in the strategies of reputation management and impression formation used *within* societies. If the reaction to any prosocial act is contingent and context-specific, then we can expect that people's exposure to and concern for social costs will vary. An individual's social standing may have particular bearing on how prosocial acts are interpreted (and so, on the individual's propensity to carry them out). However, it is not immediately clear *how* this might feed in. For example, we may expect people of lower social standing to be viewed in a positive light because of the greater relative cost they incur for any helpful act (e.g. Yuan et al., 2018). Or, we could expect the opposite, with people of lower social standing being viewed with more scepticism: if they are more in need of the potential social benefits that stem from helpful acts, then they may more readily be assumed to have strategic aims, as compared with better off individuals who could be doing things not out of need, but out of ‘the goodness of their heart’. Vignette studies could extend earlier work (Barrett et al., 2016) to determine whether and, if so, how judgments are dependent on the identity and status(es) of the actor involved. If there are indeed systematic biases in who faces derision for their prosocial acts and who garners praise, then it is also worth investigating the aggregate consequences of these inequalities. Formal and agent-based models may be particularly useful in studying the structural consequences of particular patterns of biases in interpretation.

To sum up, prosocial acts commonly yield reputation benefits, which can be advantageous in the context of partner choice. Nevertheless, there is also the potential for these investments to result in social costs – and this is most likely when observers can infer self-interested intentions or when observers believe that helpful actions have the potential to harm them. We have emphasised the contingent and context-specific nature of such evaluations. There is a clear need for more cross-cultural work in this field so that we address the extent to which some of the most frequently observed patterns are culturally specific or are more likely to be human universals.

## Data Availability

There are no data to report.
